# Trimodal Therapy in Esophageal Squamous Cell Carcinoma: Role of Adjuvant Therapy Following Neoadjuvant Chemoradiation and Surgery

**DOI:** 10.3390/cancers14153721

**Published:** 2022-07-30

**Authors:** Xiaokun Li, Siyuan Luan, Yushang Yang, Jianfeng Zhou, Qixin Shang, Pinhao Fang, Xin Xiao, Hanlu Zhang, Yong Yuan

**Affiliations:** Department of Thoracic Surgery, West China Hospital, Sichuan University, Chengdu 610000, China; drlixiaokun@163.com (X.L.); luansiyuan30@163.com (S.L.); yangysh07@163.com (Y.Y.); 2020224020107@stu.scu.edu.cn (J.Z.); qixinshang0405@gmail.com (Q.S.); 15877929137@163.com (P.F.); drxiaoxin@foxmail.com (X.X.); drzhanghanlu@wchscu.cn (H.Z.)

**Keywords:** esophageal cancer, neoadjuvant chemoradiotherapy, esophagectomy, adjuvant therapy

## Abstract

**Simple Summary:**

In this work, we aimed to explore the effectiveness of adjuvant therapy after trimodal therapy (neoadjuvant chemoradiotherapy and esophagectomy) in patients with thoracic esophageal squamous cell carcinoma (ESCC). Overall survival (OS) and disease-free survival (DFS) were both compared for adjuvant and non-adjuvant groups. Propensity score matching was used to eliminate the confounding factors between the two groups. Meanwhile, subgroup analysis based on a neoadjuvant-treated node stage (ypN) was performed to precisely stratify the patients and to guide the clinical decision-making at the point of care. As of now, there is no guideline or recommendation on the treatment of ESCC patients with adjuvant therapy after neoadjuvant chemoradiotherapy followed by surgery. The results of our study indicate that adjuvant therapy after trimodal therapy could shorten OS and DFS in patients with ESCC. Meanwhile, adjuvant therapy is an independently unfavorably prognostic factor for DFS. Therefore, adjuvant therapy is not recommended for ESCC patients after trimodal therapy, especially patients without nodal metastases after neoadjuvant therapy. To our knowledge, this is the first retrospective study using subgroup analysis to examine the effect of adjuvant therapy in ESCC patients after trimodal therapy by comparing overall survival and disease-free survival. The results of our study add useful evidence to recent guidelines.

**Abstract:**

**Background:** The aim of this study was to determine the role of adjuvant therapy after neoadjuvant chemoradiotherapy and esophagectomy for esophageal squamous cell carcinoma (ESCC). **Methods:** The study retrospectively reviewed 447 ESCC patients who underwent neoadjuvant chemoradiotherapy and esophagectomy. Patients were divided into an adjuvant therapy group and no adjuvant therapy group. Propensity score matching was used to adjust the confounding factors. **Results:** 447 patients with clinical positive lymph nodes and no distant metastasis treated with neoadjuvant chemoradiotherapy and esophagectomy were eligible for analysis. After propensity score matching, there were 120 patients remaining in each group. Patients receiving adjuvant therapy had a significantly shorter post-resection overall survival (OS) and disease-free survival (DFS) when compared to patients not receiving adjuvant therapy (log-rank, OS: *p* = 0.046, DFS: *p* < 0.001). Receiving adjuvant therapy is not an independently prognostic factor for OS (hazard ratio (HR): 1.270, HR: 0.846–1.906, *p* = 0.249) but a significantly unfavorable independent prognostic factor for DFS (HR: 2.061, HR: 1.436–2.958, *p* < 0.001). **Conclusions:** The results of our study indicate that adjuvant therapy after neoadjuvant chemoradiotherapy and surgery could reduce the OS and DFS in patients with ESCC. Therefore, adjuvant therapy is not recommended for ESCC patients after neoadjuvant chemoradiotherapy and esophagectomy, especially patients without nodal metastases after neoadjuvant therapy.

## 1. Introduction

Esophageal cancer (EC) is the sixth leading cause of cancer deaths worldwide and the second deadliest gastrointestinal cancer after gastric carcinoma [1]. The literature reports that approximately 200,000 people die of EC annually worldwide, and most cases of EC are diagnosed at advanced stages [1]. Esophageal squamous cell carcinoma (ESCC) represents the predominant subtype of EC, with most cases occurring in eastern Asia. The morbidity rate varies extremely across areas and countries [2,3].

Although a tremendous improvement of therapeutic modalities has been recently observed, patients’ quality of life remains poor, and the five-year survival rate rarely exceeds 40% [3]. Currently, surgery remains the major treatment for patients with early stage resectable ESCC, whereas neoadjuvant therapy (chemotherapy, radiotherapy, or their combination prior to surgery) followed by esophagectomy is the standard of care for those with locally advanced disease (cT1-2N+ or cT3-4aN1-3). It has been proven that patients with locally advanced esophageal cancer can benefit from trimodal therapy (neoadjuvant concurrent chemoradiation followed by surgery), when compared to surgery alone [2]. However, additional adjuvant therapy (chemotherapy and/or radiotherapy after surgery) may be necessary for patients that do not fully respond to neoadjuvant therapy, characterized by pathologically confirmed residual disease and lymph node metastasis. Nevertheless, the use of adjuvant therapy remains controversial for these patients because the therapeutic efficacy may be insufficient to control the residual disease. In addition, patients are at an additional risk of adverse events. Currently, there is no guideline recommendation to treat ESCC patients with adjuvant therapy after they receive neoadjuvant chemoradiotherapy and esophagectomy [2]. Due to a restricted number of clinical studies concerning this topic, the indication for adjuvant therapy after trimodal therapy is highly dependent on the patient and the institution [4]. Although there are several large-scale studies investigating the utility of adjuvant therapy after neoadjuvant therapy and surgery in western populations, the majority of the cases included in these cohorts are esophageal adenocarcinoma and the information regarding treatment regimens is missing [5,6,7]. Therefore, no clear evidence could guide the utilization of adjuvant therapy after trimodal therapy in patients with ESCC, especially in the east Asian region.

To add evidence to this important clinical question, we conducted a single-center and retrospective cohort study to investigate the role of adjuvant therapy following neoadjuvant chemoradiotherapy and surgery in patients with thoracic ESCC. Meanwhile, subgroup analysis based on neoadjuvant treated node stage (ypN) was performed to further explore the impact of adjuvant therapy on ESCC patients.

## 2. Materials and Methods

### 2.1. Patients

There were 447 ESCC patients undergoing neoadjuvant chemoradiotherapy and esophagectomy retrospectively reviewed at the West China Hospital from January 2014 to July 2020. The study was approved by the human participants’ committee of the West China Hospital of Sichuan University. Surgeons informed the patients concerning the risks of the neoadjuvant/adjuvant therapy and esophagectomy. The written consent of the study’s participants and permission to use resected specimens were obtained preoperatively. This study was approved by the Institutional Review Board of West China Hospital, Sichuan University in April 2021 (2022-636).

The inclusion criteria are listed as follows: (1) patients were pathologically diagnosed with ESCC before treatment, (2) patients received neoadjuvant chemoradiotherapy and esophagectomy, (3) patients were staged according to the American Joint Committee on Cancer (AJCC) 8th edition (the patients from 2014 to 2016 were staged according to AJCC 7th edition and then re-staged for the purpose of the study) [8], (4) patients were diagnosed as clinical lymph node metastasis positive (cN+) based on imaging evidence and no distant metastasis (cM0) before any treatments, (5) detailed data of the pathological information and adjuvant therapy were collected, and (6) patients were assessed as negative surgical margin pathologically after radical esophagectomy with complete tumor resection (R0 resection).

**Figure 1:**
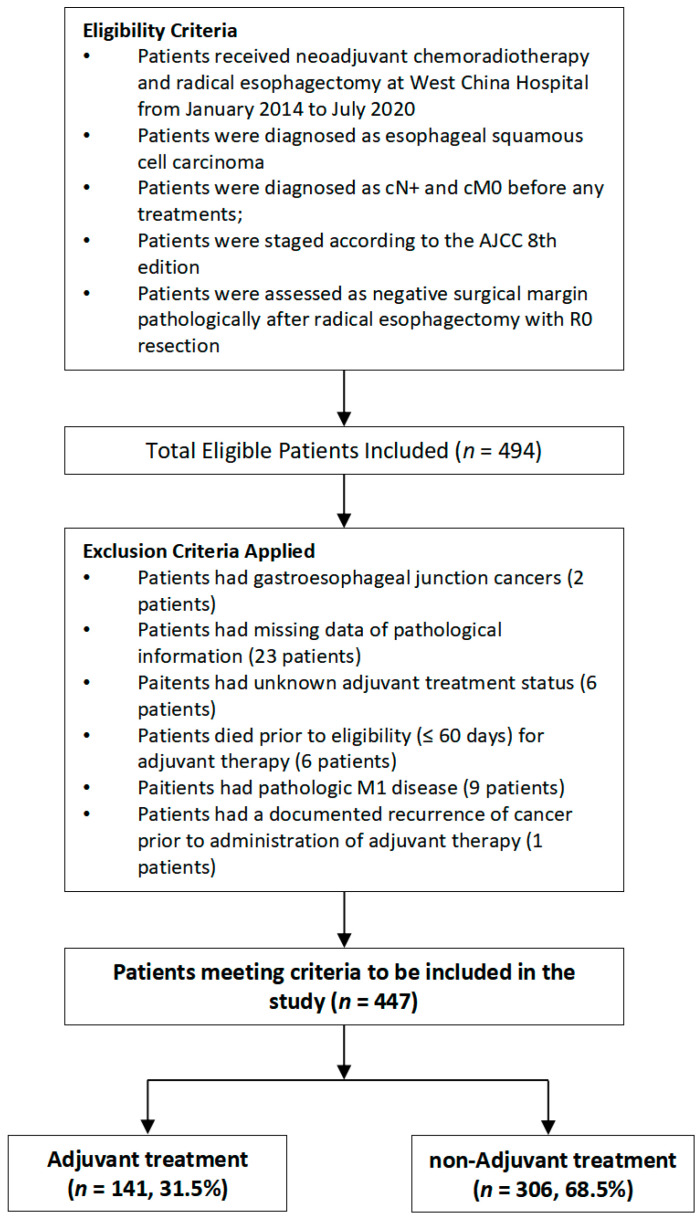
CONSORT diagram.

Patients were excluded if they had missing pathological information data, had unknown adjuvant treatment status, died prior to eligibility (≤60 days) for adjuvant therapy, had pathologic M1 disease, or had a documented recurrence of cancer prior to administration of adjuvant therapy. Only patients with ESCC were included. The CONSORT diagram (Figure 1) shows the inclusion and exclusion criteria of our study.

Patients were divided into adjuvant and non-adjuvant therapy groups for the log-rank test and Cox regression analysis. Demographic characteristics, comorbidities, operative data, postoperative complications, and pathological information were collected for all patients. Patients were followed up every 3 months for the first 2 years, and every 6 months thereafter. Neck and abdominal ultrasound, chest computerized tomography (CT), gastroscopy, and blood tests were performed on the basis of patient’s symptoms during follow-up. The patient status (including death and survival), the tumor status (including tumor recurrence and metastasis), and the patient loss of follow-up were all documented. Our follow-ups were implemented via telephone or outpatient department visit. The last follow-up was conducted on 1 January 2022.

### 2.2. Neoadjuvant Therapy

The selection of neoadjuvant therapy depended on the preoperative clinical stage of the ESCC patients. For patients with cN1-3 and/or cT4a-b, neoadjuvant chemoradiotherapy was routinely administered. The chemotherapeutic drugs were selected according to National Comprehensive Cancer Network (NCCN) Guidelines for esophageal and esophagogastric junction cancers and previous publications [2,9,10]. Neoadjuvant chemoradiotherapy included two cycles of chemotherapy with sequential or concurrent radiotherapy. The neoadjuvant chemoradiotherapy treatment cycle was 21 days (treatment during weeks 1 and 4). Paclitaxel (China Shiyao Pharmaceutical Group Co., Ltd., Shijiazhuang, China) in a dose of 175 mg/m^2^ (day 1) or carboplatin (Qilu Pharmaceutical Group Co., Ltd., Jinan, China) in a dose of area under the concentration–time curve 5 (day 1), with a combination of cisplatin (Qilu Pharmaceutical Group Co., Ltd., Jinan, China) in the amount of 75 mg/m^2^/24 h (days 1–2 or days 1–3), was given intravenously. Patients received concurrent radiation up to a total dose of 40–50.4 gray (Gy), delivered in 1.8–2.0 Gy fractions, beginning on day 1 of the first chemotherapy cycle (week 1) and ending at the completion of the second chemotherapy cycle (week 4). Sequential radiation to the same doses was arranged after the end of the second chemotherapy cycle. Intensity-modulated radiotherapy technique was used to perform radiotherapy in all patients. We referred to the Ryan scoring system to score tumor regression grades (TRGs) [11]. TRGs 0–3 are defined as follows: TRG 0: complete response (no viable cancer cells), TRG 1: near complete response (rare small groups of cancer cells), TRG 2: partial response (residual cancer with evident tumor regression), and TRG 3: poor or no response (extensive residual cancer with no evident tumor regression). Three pathologists reexamined the results of the pathological sections, and the final TRG had to be agreed upon by two or more pathologists. The strategy of neoadjuvant chemoradiotherapy is showed in Table S1.

### 2.3. Surgical Procedure and Pathology

McKeown esophagectomy with cervical anastomoses or Ivor Lewis esophagectomy with thoracic anastomoses combined with radical lymph node dissection was performed in a standardized manner. The gastric conduit was used to reconstruct the upper digestive tract during esophagectomy. The lymph nodes were then separated by surgeons from the dissected peri-esophagus and esophagus tissues. Specimens were sent to the pathology department for further analysis where representative sections of the tumor and periesophageal tissues were taken for sufficient pathologic evaluation and staging.

### 2.4. Adjuvant Therapy

In our institution, each patient was evaluated by a multidisciplinary team by whom adjuvant therapy selection was determined. The final decision was left up to the patients’ preference. The adjuvant chemotherapeutic regimens were selected according to the NCCN Guidelines for esophageal and esophagogastric junction cancers and previous publications [2,9,12]. Generally, the chemotherapy regimens included 5-fluorouracil and cisplatin, repeated twice every 3 weeks. 5-fluorouracil in a dose of 800 mg/m^2^ was given by continuous infusion on days 1 through 5. Cisplatin in a dose of 80 mg/m^2^ was administered by intravenous drip infusion for 2 h on day 1. An intensity-modulated radiotherapy technique was used to administer radiotherapy with a total dose of 45 to 50.4 Gy (1.8–2.0 Gy/d). Combined chemoradiotherapy included giving radiotherapy from the first day of the first chemotherapy cycle. Two cycle of Tislelizumab (200 mg, D1), Sintilimab (200 mg, D1), or Pembrolizumab (200 mg, D1) administered by intravenous injection combined with radiotherapy was implemented for patients undergoing adjuvant immune radiotherapy. The immunotherapy was repeated twice every 3 weeks. Typically, adjuvant therapy is administered 4 to 6 weeks after esophagectomy based on NCCN Guideline [2,12]. The strategy for adjuvant therapy is showed in Table S2.

### 2.5. Statistical Analysis

Pearson’s chi-square test was used to compare categorical variables expressed as frequencies. Kaplan-Meier curves were used to analyze overall survival (OS) and disease-free survival (DFS), and the log-rank test was employed to determine statistical significance between the adjuvant and non-adjuvant therapy groups. A Cox regression model was used to determine variables independently associated with OS and DFS for patients undergoing neoadjuvant therapy and esophagectomy. Variables were selected for multivariate Cox regression model entry if *p* < 0.05 on univariate analysis. In addition, hazard ratios with 95% confidence intervals (CI) were reported, and we assessed whether the treatment effect differed in certain subgroups by testing the treatment-by-subgroup interaction effect with the use of Cox models via univariate analysis. All tests were two-sided and *p* < 0.05 was considered to be statistically significant. All statistical analyses were implemented with R (version 3.5.3). SPSS version 27.0 software (SPSS Inc. Chicago, IL, USA) was used to perform propensity score matching. The confounding factors including gender, age, smoke history, tumor length, neoadjuvant treated tumor, node, and metastases (ypTNM) stage, neoadjuvant treated tumor (ypT) status, ypN status, tumor differentiation, lymphovascular invasion, peripheral nerve invasion, and tumor regression grade were employed to develop the propensity score matching. The nearest-neighbor method with a caliper width of 0.02 was used to match the selected cases from two groups at a ratio of 1:1.

## 3. Results

### 3.1. Patient Characteristics

After application of the inclusion and exclusion criteria, 447 patients with cN+ and cM0 following neoadjuvant chemoradiotherapy and radical esophagectomy were available for analysis. Demographic characteristics, comorbidities, operative data, postoperative complications, and pathological information of the included patients are displayed in Table 1. The median tumor length was 3 cm, which was used as the cut-off value. A complete response (TRG 0) was reported in 150 (33.6%) patients, a near complete response (TRG 1) in 73 (16.3%) patients, a partial response (TRG 2) in 170 (38.0%) patients, and a poor or non-response (TRG 3) in 68 (13.4%) patients. Adjuvant therapy was performed in 141 (31.5%) patients. Of these, 49 (34.8%) received adjuvant chemotherapy, 15 (10.6%) received adjuvant radiotherapy alone, 40 (28.4%) received chemoradiotherapy, and 37 (26.2%) received immuno-radiotherapy. A total of 306 (68.5%) patients received no adjuvant therapy. Patients receiving adjuvant therapy were more likely to have a younger age, a history of smoking, an upper tumor site, a poorer tumor stage, more positive lymph nodes, advanced stage, increased lymphovascular and peripheral nerve invasion, and poorer response to neoadjuvant therapy. Due to the heterogeneity between the two groups, propensity score matching was used to balance the baseline characteristics between the adjuvant group and the non-adjuvant group. After propensity score matching, there were 120 patients remaining in each group and the patients were adjusted for all the potential confounding factors (Table 1). After propensity score matching, there were 38 (31.7%) patients receiving adjuvant chemotherapy, 13 (10.8%) receiving adjuvant radiotherapy alone, 35 (29.2%) receiving chemoradiotherapy, and 34 (28.3%) receiving immuno-radiotherapy.

### 3.2. Survival Analysis

**Figure 2:**
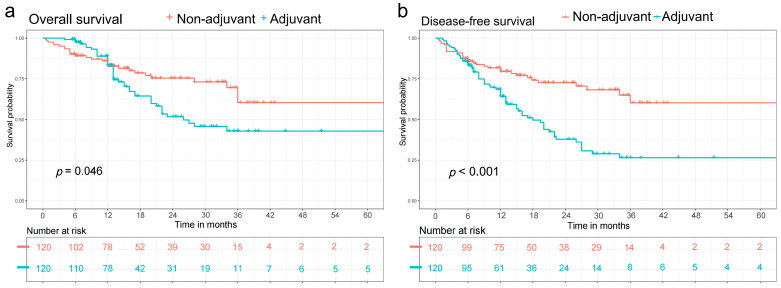
After propensity score matching, Kaplan-Meier curves were used to analyze overall survival (OS) and disease-free survival (DFS), and the log-rank test was employed to determine statistical significance between the two groups. (**a**) Comparison of OS between patients receiving and not receiving adjuvant therapy. Patients receiving adjuvant therapy had a shorter post-resection OS compared with patients not receiving adjuvant therapy (log-rank, OS: *p* = 0.046) (**b**) Comparison of DFS between patients receiving and not receiving adjuvant therapy. Patients receiving adjuvant therapy also had a shorter post-resection DFS compared with patients not receiving adjuvant therapy (log-rank, DFS: *p* < 0.001).

The median follow-up time was 13.4 months (interquartile range 6.7–24.47 months) for the overall cohort, 13.38 months (6.8–24.39 months) for those who received adjuvant therapy, and 13.43 months (6.3–25.3 months) for those who did not. After propensity score matching, patients that received adjuvant therapy had a shorter post-resection OS compared to patients not receiving adjuvant therapy (log-rank, OS: *p* = 0.046 (Figure 2a)). Meanwhile, patients receiving adjuvant therapy also had a shorter post-resection DFS compared with patients not receiving adjuvant therapy (log-rank, DFS: *p* < 0.001 (Figure 2a)).

**Figure 3:**
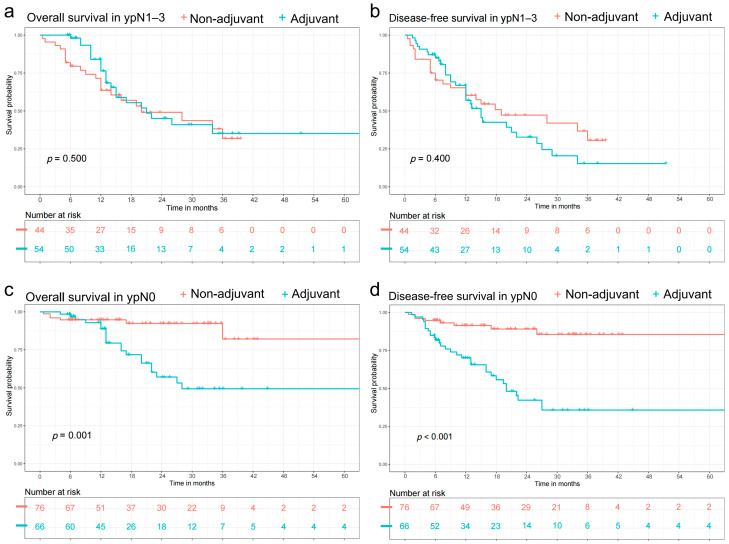
After propensity score matching, Kaplan-Meier curves were used to analyze overall survival (OS) and disease-free survival (DFS), and the log-rank test was employed to determine statistical significance between groups. Subgroup survival analysis were performed stratified by the neoadjuvant treated node (ypN) stage (**a**) In the patients with ypN1–3, there was no significant difference in OS between two groups (*p* = 0.500) (**b**) In the patients with ypN1–3, there was no significant difference in DFS between two groups (*p* = 0.400) (**c**) In the patients with ypN0, the adjuvant therapy group yielded a significantly shorter OS compared with non-adjuvant therapy group (*p* = 0.001) (**d**) In the patients with ypN0, the adjuvant therapy group yielded a significantly shorter DFS compared with non-adjuvant therapy group (*p* < 0.001).

Subgroup survival analysis was performed stratified by the ypN stage (Figure 3). Among the patients with ypN1–3, there was no significant difference in OS between the adjuvant and non-adjuvant groups (*p* = 0.500) (Figure 3a). Meanwhile, no significant difference was found in DFS for patients with ypN1–3 (*p* = 0.400) (Figure 3b). When comparing the OS between the two groups in patients with ypN0, the adjuvant therapy group had a significantly shorter OS when compared with non-adjuvant therapy group (*p* = 0.001) (Figure 3c). Meanwhile, for ypN0 patients the adjuvant therapy group also had a significantly shorter DFS compared to the non-adjuvant therapy group (*p* < 0.001) (Figure 3d).

### 3.3. Cox Regression Analysis

There were 13 variables included in the univariate Cox regression model (Table S3). Eight variables were selected for multivariate Cox regression model entry due to *p* < 0.05 on univariate analysis (Table 2). The results of Cox regression analysis on OS shows that only the ypTNM stage was an independent prognostic factor for OS in patients undergoing neoadjuvant therapy and esophagectomy. However, receiving adjuvant therapy was not an independent prognostic factor for OS (hazard ratio (HR): 1.270, 95% CI: 0.846–1.906, *p* = 0.249). The results of the Cox regression analysis on DFS show that ypTNM stage and adjuvant therapy were independent prognostic factors for DFS patients undergoing neoadjuvant therapy and esophagectomy. Meanwhile, receiving adjuvant therapy was a significantly unfavorably independent prognostic factor for DFS (HR: 2.061, 95% CI: 1.436–2.958, *p* < 0.001).

### 3.4. Subgroup Analysis by Forest Plot

**Figure 4:**
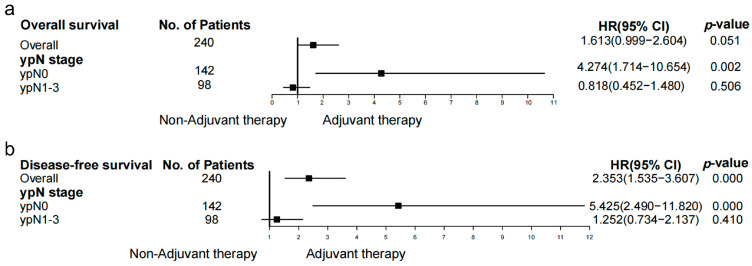
Subgroup analysis with Cox regression model. (**a**) Hazard ratios with 95% CI for the overall survival in prespecified subgroups. For patients with ypN0, adjuvant therapy is an unfavorable factor for OS (HR: 4.274, 95% CI: 1.714–10.654, *p* = 0.002). For patients with ypN1–3, adjuvant therapy is not a prognostic factor for OS (HR: 0.818, 95% CI: 0.452–1.480, *p* = 0.506). (**b**) Hazard ratios with 95% CI for the disease-free survival in prespecified subgroups. For patients with ypN0, adjuvant therapy is an unfavorable factor for DFS (HR: 5.425, 95% CI: 2.490–11.820, *p* < 0.001). For patients with ypN1–3, adjuvant therapy is not a prognostic factor for DFS (HR: 1.252, 95% CI: 0.734–2.137, *p* = 0.410).

Figure 4 shows the hazard ratios with 95% CIs for the OS outcome in prespecified subgroups. According to the results of the overall analysis, adjuvant therapy was not a prognostic factor for OS (HR: 1.613, 95% CI: 0.999–2.604, *p* = 0.051). However, adjuvant therapy was an unfavorable prognostic factor for DFS (HR: 2.353, 95% CI: 1.535–3.607, *p* < 0.001). In subgroup analysis, for patients with ypN0, adjuvant therapy was an unfavorable factor for OS (HR: 4.274, 95% CI: 1.714–10.654, *p* = 0.002) and DFS (HR: 5.425, 95% CI: 2.490–11.820, *p* < 0.001). Nevertheless, for patients with ypN1–3, adjuvant therapy was not a prognostic factor for OS (HR: 0.818, 95% CI: 0.452–1.480, *p* = 0.506) or DFS (HR: 1.252, 95% CI: 0.734–2.137, *p* = 0.410). Table 3 contains brief information on the outcomes of prior high-quality publications and the present study.

## 4. Discussion

In this retrospective study, we evaluated the effectiveness of adjuvant therapy on ESCC patients treated with neoadjuvant therapy and surgery. Concurrent neoadjuvant chemoradiotherapy followed by surgery has been considered as a preferred treatment strategy for patients diagnosed as ESCC in China [16,17]. However, a guideline regarding the use of adjuvant therapy after trimodal therapy in patients with ESCC is still missing. According to NCCN guidelines, the use of adjuvant therapy is recommended for all patients with esophageal adenocarcinoma after trimodal therapy, regardless of the existence of positive lymph nodes and pathologic response [2]. However, on account of different epidemiological characteristics it remains unclear if ESCC patients can benefit from adjuvant therapy. Therefore, we conducted this retrospective study to explore the effect of adjuvant therapy after neoadjuvant chemoradiotherapy and surgery in ESCC patients. Meanwhile, subgroup analysis was performed to precisely stratify the patients undergoing neoadjuvant therapy followed by esophagectomy and to provide clinical evidence that can be utilized to guide the multimodal care of ESCC patients.

Burt et al. [7] first conducted a large-scale retrospective study based on data from the National Cancer Database (NCDB) to investigate the role of adjuvant therapy after trimodal therapy in patients diagnosed as EC. Their study indicated that EC patients with residual nodal disease after treatment with neoadjuvant chemoradiation could benefit from adjuvant chemotherapy. However, this benefit cannot be found in patients with no residual nodal disease. Whereafter, Samson et al. [6] reported a retrospective cohort study based on NCDB data only including patients with pathologic node-positive EC after neoadjuvant chemotherapy. Their study came to the same conclusion as the study reported by Burt et al. [7]. In the same year, Mokdad et al. [5] explored the effect of adjuvant chemotherapy after trimodal therapy in patients with gastroesophageal adenocarcinoma. They concluded that patients with gastroesophageal adenocarcinoma could obtain a survival benefit from adjuvant chemotherapy after trimodal therapy regardless of pathologic node status. In 2019, Drake et al. [13] investigated the effect of adjuvant chemotherapy after neoadjuvant therapy and esophagectomy. Their study only included esophageal adenocarcinoma patients with nodal metastases and concluded with the same findings as the study reported by Mokdad et al. [5]. Thereafter, Semenkovich et al. [14] conducted a multicenter retrospective cohort study including both ESCC and esophageal adenocarcinoma patients with nodal metastases, which showed that the patients with pathologic node-positive EC could benefit from adjuvant therapy after neoadjuvant therapy and surgery. Huang et al. [15] conducted a retrospective cohort study including 228 ESCC patients to investigate the effect of adjuvant therapy after neoadjuvant chemotherapy and surgery in 2019. The results of their study showed no significant difference in OS or DFS between the adjuvant therapy group and the non-adjuvant therapy group after propensity score matching. However, subgroup analysis based on status of nodal metastases were not implemented.

In our study, we only included patients with thoracic ESCC. Meanwhile, propensity score matching was used to eliminate the confounding factors, which makes the results more reliable. The results indicated that patients undergoing adjuvant therapy after trimodal therapy yielded significantly shorter OS and DFS when compared to patients not receiving adjuvant therapy. The results were consistent in patients with pathologic node-negative ESCC. However, for patients with nodal metastases after neoadjuvant chemoradiotherapy, no significant difference was seen between adjuvant therapy groups and no adjuvant therapy group. The results were opposite to the study reported by Matsuura et al. [18]. They conducted a retrospective study enrolling 113 thoracic ESCC patients with three or more pathologic positive lymph nodes. The included patients received neoadjuvant chemotherapy followed by radical surgery. The clinical efficacy of adjuvant chemotherapy was then evaluated. Their study concluded that adjuvant therapy may offer a significantly additional benefit to the prognosis of EC patients who have many positive lymph nodes even after neoadjuvant chemotherapy and surgery. The potential reason for their different results could be that their study only included patients with three or more pathologic positive lymph nodes (ypN2–3). However, the evidence was not irrefutable due to the small study population.

Theoretically, adjuvant therapy is expected to instigate a favorable effect and prolong survival for patients. However, in our institution adjuvant therapy could not prolong survival for patients with ESCC after trimodal therapy, even for patients with pathologic positive lymph nodes (ypN1–3). On the contrary, for ESCC patients with pathologic negative lymph nodes (ypN0), adjuvant therapy could be an unfavorable prognostic factor. There are several potential explanations for these results. Patients who were already treated with preoperative chemoradiotherapy could be insensitive to repeated systemic treatment after surgery [19,20]. Meanwhile, as a systemic treatment, all types of adjuvant therapy may cause adverse systemic effects on patients [21]. Especially for ESCC patients, prolonged fasting or reduce of meal could lead to poor nutritional status, which makes them frailer after receiving adjuvant therapy [22,23]. Moreover, the unfavorable impact of adjuvant therapy on the immune system could further weaken the patient’s resistance to the tumor, leading to tumor recurrence after adjuvant therapy [24]. Therefore, adjuvant therapy is not recommended for ESCC patients after trimodal therapy regardless of status of nodal metastases.

There are several limitations present in this study. The retrospective nature of this study design could reduce the reliability of the results. Therefore, propensity score matching was used in this study to eliminate the selection bias of included patients. Meanwhile, the sample size is small because of the single-center setting. More participants will be employed in our future study.

## 5. Conclusions

The results of our study indicate that adjuvant therapy after neoadjuvant chemoradiotherapy and surgery could reduce the OS and DFS in patients with ESCC. Meanwhile, adjuvant therapy is an independently unfavorably prognostic factor for DFS. Therefore, adjuvant therapy is not recommended for ESCC patients after neoadjuvant chemoradiotherapy and esophagectomy, especially patients with node-negative after neoadjuvant therapy. A large-scale well-designed prospective study will be needed to confirm these results.

## Figures and Tables

**Table 1:** Baseline characteristics of the patients.

Variable	No. (%) (*n* = 447)	Before Propensity Score Match			After Propensity Score Match		
		Non-Adjuvant Therapy (*n* = 306)	Adjuvant Therapy (*n* = 141)	*p*-Value	Non-Adjuvant Therapy (*n* = 120)	Adjuvant Therapy (*n* = 120)	*p*-Value
**Gender**				0.155			0.701
Male	359 (80.3%)	274 (79.9%)	121 (85.8%)		106 (88.3%)	103 (85.8%)	
Female	88 (19.7%)	69 (20.1%)	20 (14.2%)		14 (11.6%)	17 (14.2%)	
**Age (year)**				0.004			0.331
≤65	287 (64.2%)	183 (59.8%)	104 (73.8%)		78 (65.0%)	86 (71.7%)	
>65	160 (35.8%)	123 (40.2%)	37 (26.2%)		42 (35.0%)	34 (28.3%)	
**Smoke**				0.014			0.155
Yes	230 (51.5%)	145 (47.4%)	85 (60.3%)		58 (48.3%)	70 (58.3%)	
No	217 (48.5%)	161 (52.6%)	56 (39.7%)		62 (51.7%)	50 (41.7%)	
**Alcohol consumption**				0.837			0.517
Yes	187 (41.8%)	127 (41.5%)	60 (42.6%)		52 (43.3%)	57 (47.5%)	
No	260 (58.2%)	179 (58.5%)	81 (57.4%)		68 (56.7%)	63 (52.5%)	
**Hypertension**				0.895			0.869
Yes	80 (17.9%)	54 (17.6%)	26 (18.4%)		23 (19.2%)	22 (18.3%)	
No	367 (82.1%)	252 (82.4%)	115 (81.6%)		97 (80.8%)	98 (81.7%)	
**Cardiovascular disease** (*n* = 444)				0.450			0.518
Yes	19 (4.3%)	15 (4.9%)	4 (2.9%)		6 (5.0%)	4 (3.3%)	
No	425 (95.7%)	290 (95.1%))	135 (97.1%)		114 (95.0%)	116 (96.7%)	
**Cerebrovascular disease** (*n* = 442)				0.443			0.999
Yes	7 (1.6%)	6 (2.0%)	1 (0.7%)		1 (0.8%)	1 (0.8%)	
No	435 (98.4%)	298 (98.0%)	137 (99.3%)		119 (99.2%)	119 (99.2%)	
**Chronic liver disease** (*n* = 434)				0.853			0.999
Yes	37 (8.5%)	25 (8.3%)	12 (9.0%)		10 (8.3%)	10 (8.3%)	
No	397 (91.5%)	275 (91.7%)	122 (91.0%)		110 (91.7%)	110 (91.7%)	
**COPD** (*n* = 444)				0.533			0.554
Yes	28 (6.3%)	21 (6.9%)	7 (5.0%)		7 (5.8%)	5 (4.2%)	
No	416 (93.7%)	284 (93.1%)	132 (95.0%)		113 (94.2%)	115 (95.8%)	
**Arrhythmia** (*n* = 446)				0.515			0.651
Yes	10 (2.2%)	8 (2.6%)	2 (1.4%)		3 (2.5%)	2 (1.7%)	
No	436 (97.8%)	297 (97.4%)	139 (98.6%)		117 (97.5%)	118 (98.3%)	
**Tumor site**				0.046			0.383
Upper	61 (13.6%)	34 (11.1%)	27 (19.1%)		11 (9.2%)	18 (15.0%)	
Middle	229 (51.2%)	160 (52.3%)	69 (48.9%)		63 (52.5%)	59 (49.2%)	
Lower	157 (35.1%)	112 (36.6%)	45 (31.9%)		46 (38.3%)	43 (35.8%)	
**Tumor length (cm)**				0.001			0.694
≤3	289 (64.7%)	216 (70.6%)	73 (51.8%)		72 (60.0%)	69 (57.5%)	
>3	158 (35.3%)	90 (29.4%)	68 (48.2%)		48 (40.0%)	51 (42.5%)	
**ypTNM**				0.000			0.160
I	219 (49.0%)	174 (56.9%)	45 (31.9%)		57 (47.5%)	43 (35.8%)	
II	64 (14.3%)	40 (13.1%)	24 (17.0%)		19 (15.8%)	22 (18.3%)	
IIIA	55 (12.3%)	34 (11.1%)	21 (14.9%)		9 (7.5%)	18 (15.0%)	
IIIB	96 (21.5%)	50 (16.3%)	46 (32.6%)		29 (24.2%)	34 (28.3%)	
IVA	13 (2.9%)	8 (2.6%)	5 (3.5%)		6 (5.0%)	3 (2.5%)	
**ypT**				0.001			0.493
Tis	2 (0.4%)	2 (0.6%)	0 (0.0%)		0 (0.0%)	0 (0.0%)	
T0	161 (36.0%)	127 (41.5%)	34 (24.1%)		42 (35.0%)	32 (26.6%)	
T1	64 (14.3%)	44 (14.4%)	20 (14.2%)		12 (10.0%)	17 (14.2%)	
T2	65 (14.5%)	44 (14.4%)	21 (14.9%)		19 (15.8%)	20 (16.7%)	
T3	155 (34.5%)	89 (28.8%)	66 (46.8%)		47 (39.2%)	51 (42.5%)	
**ypN**				0.001			0.304
N0	284 (63.5%)	214 (69.9%)	70 (49.6%)		76 (63.3%)	66 (55.0%)	
N1	112 (25.1%)	67 (21.9%)	45 (31.9%)		26 (21.7%)	34 (28.3%)	
N2	39 (8.7%)	18 (5.9%)	21 (14.9%)		12 (10.0%)	17 (14.2%)	
N3	12 (2.7%)	8 (2.6%)	5 (3.5%)		6 (5.0%)	3 (2.5%)	
**Tumor differentiation**				0.000			0.116
G1	13 (2.9%)	10 (3.3%)	3 (2.1%)		5 (4.2%)	2 (1.7%)	
G2	108 (24.2%)	73 (23.9%)	35 (24.8%)		35 (29.2%)	25 (20.8%)	
G3	138 (30.9%)	77 (25.2%)	61 (43.3%)		37 (30.8%)	53 (44.2%)	
Gx	188 (42.1%)	146 (47.7%)	42 (29.8%)		43 (35.8%)	40 (33.3%)	
**Lymphovascular invasion**				0.001			0.336
Yes	42 (9.4%)	19 (6.2%)	23 (16.3%)		13 (10.8%)	18 (15.0%)	
No	405 (90.6%)	287 (93.8%)	118 (83.7%)		107 (89.2%)	102 (85.0%)	
**Peripheral nerve invasion**				0.002			0.525
Yes	80 (17.9%)	43 (14.1%)	37 (26.2%)		23 (19.2%)	27 (22.5%)	
No	367 (82.1%)	263 (85.9%)	104 (73.8%)		97 (80.8%)	93 (77.5%)	
**Surgical type**				0.198			0.678
Open surgery	45 (10.1%)	27 (8.8%)	18 (12.8%)		14 (11.7%)	12 (10.0%)	
Video-assisted Thoracoscopic Surgery	402 (89.9%)	279 (91.2%)	123 (87.2%)		106 (88.3%)	108 (90.0%)	
**Anastomotic method**				0.285			0.313
Stapled anastomosis	16 (3.6%)	9 (2.9%)	7 (5.0%)		3 (2.5%)	6 (5.0%)	
Hand-sewn anastomosis	431 (96.4%)	297 (97.1%)	134 (95.0%)		117 (97.5%)	114 (95.0%)	
**Complications (Clavien-Dindo)**				0.606			0.619
Grade I	73 (16.3%)	47 (15.4%)	26 (18.4%)		17 (41.2%)	22 (18.3%)	
Grade II	149 (33.3%)	104 (34.0%)	45 (31.9%)		40 (33.3%)	39 (32.5%)	
Grade III	29 (6.5%)	21 (6.9%)	8 (5.7%)		12 (10.0%)	8 (6.7%)	
Grade IV	7 (1.6%)	6 (2.0%)	1 (0.7%)		2 (1.7%)	1 (0.8%)	
**Tumor regression grade**				0.000			0.451
TRG 0	150 (33.6%)	120 (39.2%)	30 (21.3%)		39 (32.5%)	28 (23.3%)	
TRG 1	73 (16.3%)	55 (18.0%)	18 (12.8%)		15 (12.5%)	17 (41.2%)	
TRG 2	170 (38.0%)	101 (33.0%)	69 (48.9%)		51 (42.5%)	56 (46.7%)	
TRG 3	54 (12.1%)	30 (9.8%)	24 (17.0%)		15 (12.5%)	19 (15.8%)	

Pearson’s chi-squared test was used to compare categorical variables expressed as frequencies. An independent-sample Student’s *t*-test was used to compare continuous variables. COPD, chronic obstructive pulmonary disease; ypTNM, neoadjuvant-treated TNM; ypT, neoadjuvant-treated tumor stage; ypN, neoadjuvant-treated node stage; TRG, tumor regression grade.

**Table 2:** Cox regression model for variables independently associated with adjuvant therapy status for patients with positive nodal disease after neoadjuvant chemoradiotherapy and radical esophagectomy. Ten variables were selected for multivariate Cox regression model entry due to *p* < 0.05 in univariate analysis.

	Overall Survival			Progression-Free Survival		
Multivariate Analyses	HR	95% CI of HR	*p*-Value	HR	95% CI of HR	*p*-Value
**Gender**						
Male versus female	1.034	0.533–2.005	0.921	1.004	0.562–1.794	0.990
**Smoke**						
Yes versus no	1.505	0.917–2.467	0.106	1.490	0.961–1.924	0.075
**Tumor length**						
>3 cm versus ≤3 cm	1.486	0.989–2.234	0.056	1.346	0.941–1.924	0.103
**ypTNM**						
III-IV versus I-II	2.720	1.741–4.249	0.000	2.079	1.411–3.065	0.000
**Lymphovascular invasion**						
Yes versus no	1.095	0.626–1.915	1.095	1.324	0.819–2.140	0.251
**Peripheral nerve invasion**						
Yes versus no	0.912	0.558–1.490	0.712	1.409	0.919–2.159	0.115
**Tumor regression grade**						
TRG 3/2 versus TRG 1/0	1.358	0.839–2.198	0.212	1.074	0.703–1.640	0.743
**Adjuvant Therapy**						
Yes versus no	1.270	0.846–1.906	0.249	2.061	1.436–2.958	0.000

Cox regression model was used to determine variables independently associated with OS and DFS for patients undergoing neoadjuvant therapy and esophagectomy. ypTNM, neoadjuvant treated TNM; TRG, tumor regression grade.

**Table 3:** Previous publications evaluating the therapeutic value of adjuvant therapy following neoadjuvant therapy and esophagectomy.

Study	Year	Design	Sample Size	Histological Type	ypN Stage	Hazard Ratio	*p* Value
Burt BM, et al. [7]	2017	Retrospective cohort study based on NCDB	3592	EAC, ESCC	Any	0.93 (ypN0)	Not significant
						0.7 (ypN1–3)	Significant
Samson P, et al. [6]	2018	Retrospective cohort study based on NCDB	3100	EAC, ESCC	+	0.69	<0.001
Mokdad AA, et al. [5]	2018	Retrospective cohort study based on NCDB	10,086	Gastroesophageal adenocarcinoma	Any	0.79	<0.001
						0.68 (ypN0)	Significant
						0.86 (ypN1–3)	Significant
Drake J, et al. [13]	2019	Retrospective cohort study based on NCDB	2046	EAC	+	0.839	0.0311
Semenkovich TR, et al. [14]	2019	Multicenter retrospective cohort study	1082	EAC, ESCC	+	0.76	0.005
Huang Z, et al. [15]	2019	Retrospective cohort study	228	ESCC	Any	1.498	0.052
The present study	2022	Retrospective cohort study	447	ESCC	Any	1.613	0.051
						4.274 (ypN0)	0.002
						0.818 (ypN1–3)	0.506

NCDB, National Cancer Database; EAC, esophageal adenocarcinoma; ESCC, esophageal squamous cell carcinoma; ypN, neoadjuvant treated node status.

## Data Availability

The datasets generated during and/or analyzed during the current study are available from the corresponding author on reasonable request.

## Supplementary Materials

The following supporting information can be downloaded at: https://www.mdpi.com/article/10.3390/cancers14153721/s1, Table S1: Treatment protocol of neoadjuvant therapy; Table S2: Treatment protocol of adjuvant therapy. Table S3. Univariate Cox regression model was used to determine variables associated with overall survival and disease-free survival for patients undergoing neoadjuvant therapy and esophagectomy.
