# Supplementary material for: Trimodal Therapy in Esophageal Squamous Cell Carcinoma: Role of Adjuvant Therapy Following Neoadjuvant Chemoradiation and Surgery
Source: Cancers (Basel). 2022 Jul 30;14(15):3721. doi: 10.3390/cancers14153721 (PMC9367572; doi:10.3390/cancers14153721)
Supplement: Supplementary file 1 [file cancers-14-03721-s001.zip › cancers-1797150-supplementary.pdf]

Table S1. Treatment protocol of neoadjuvant therapy.

Neoadjuvant therapy	Regimen/technique	Dose	Schedule
Chemotherapeutic agent 1	Paclitaxel	175 mg/m^2	q3w, 2 cycles
	Carboplatin	AUC 5	q3w, 2 cycles
Chemotherapeutic agent 2	Cisplatin	75 mg/m^2	q3w, 2 cycles
Radiotherapy	Intensity-modulated radiotherapy technique	40-50.4 Gy (1.8-2.0 Gy per fraction)	Concurrent or sequential

Table S2. Treatment protocol of adjuvant therapy.

Adjuvant therapy	Regimen/technique	Dose	Schedule
Chemotherapeutic agent	5-fluorouracil	800 mg/m2	q3w, 2 cycles
	Cisplatin	80 mg/m2	q3w, 2 cycles
Radiotherapy	Intensity-modulated radiotherapy technique	40-50.4 Gy (1.8-2.0 Gy per fraction)	Concurrent or sequential
Immunotherapeutic agent 1	Tislelizumab	200mg	q3w, 2 cycles
Immunotherapeutic agent 2	Sintilimab	200mg	q3w, 2 cycles
Immunotherapeutic agent 3	Pembrolizumab	200mg	q3w, 2 cycles

Table S3. Univariate Cox regression model was used to determine variables associated with overall survival and disease-free survival for patients undergoing neoadjuvant therapy and esophagectomy.

	Overall Survival			Progression-Free Survival		
**Univariate analyses**	**HR**	**95% CI of HR**	***p*-Value**	**HR**	**95% CI of HR**	***p*-Value**
**Gender**						
Male versus female	1.736	1.020-2.953	0.042	1.638	1.028-2.609	0.038
**Age (year)**						
> 65 versus ≤ 65	1.119	0.911-1.376	0.284	1.131	0.942-1.357	0.187
**Smoke**						
Yes versus No	1.712	1.153-2.543	0.008	1.699	1.198-2.410	0.003
**Alcohol consumption**						
Yes versus No	0.978	0.602-1.590	0.929	0.993	0.644-1.532	0.975
**Preoperative complication**						
Yes versus No	0.431	0.106-1.750	0.239	0.755	0.308-1.849	0.538
**Tumor site**						
Middle/Lower versus Upper	0.956	0.545-1.677	0.875	0.915	0.563-1.486	0.719
**Tumor length**						
> 3cm versus ≤ 3cm	2.128	1.458-3.106	0.000	2.034	1.457-2.841	0.000
**ypTNM**						
III-IV versus I-II	3.686	2.497-5.441	0.000	3.098	2.207-4.349	0.000
**Tumor differentiation**						
G3/Gx versus G2/G1	0.939	0.625-1.412	0.763	0.886	0.618-1.269	0.509
**Lymphovascular invasion**						
Yes versus No	2.126	1.264-3.576	0.004	2.712	1.741-4.225	0.000
**Peripheral nerve invasion**						
Yes versus No	1.665	1.070-2.591	0.024	2.214	1.520-3.226	0.000
**Tumor regression grade**						
TRG 3/2 versus TRG 1/0	2.515	1.673-3.781	0.000	2.256	1.585-3.212	0.000
**Adjuvant Therapy**						
Yes versus No	2.076	1.429-3.016	0.000	3.029	2.166-4.237	0.000

Cox regression model was used to determine variables associated with overall survival and disease-free survival for patients undergoing neoadjuvant therapy and esophagectomy. ypTNM: neoadjuvant treated TNM; TRG: Tumor regression grade
